# Optimizing Palliative Pelvic Radiotherapy in Gynecological Cancers: A Systematic Review and Analysis

**DOI:** 10.3390/diagnostics14050547

**Published:** 2024-03-05

**Authors:** Beatrice Anghel, Mihai-Teodor Georgescu, Crenguta Sorina Serboiu, Andreea Nicoleta Marinescu, Cătălin Aliuș, Dragoș-Eugen Georgescu, Bogdan Mocanu, Sabina Sucuri, Anca Daniela Stanescu

**Affiliations:** 1 Faculty of Medicine, “Carol Davila” University of Medicine and Pharmacy, 020021 Bucharest, Romania; beatrice.dragomir-anghel@drd.umfcd.ro (B.A.); stanescuancadaniela@yahoo.com (A.D.S.); 2 Prof. Dr. Al. Trestioreanu Oncology Discipline, Faculty of Medicine, “Carol Davila” University of Medicine and Pharmacy, 022328 Bucharest, Romania; 3 Department of Histology, Carol Davila University of Medicine and Pharmacy, 020021 Bucharest, Romania; sorina.serboiu@umfcd.ro; 4 Radiology and Imaging Department, Carol Davila University of Medicine and Pharmacy, 020021 Bucharest, Romania; 5 General Surgery Department, Emergency University Hospital Bucharest, 050098 Bucharest, Romania; alius.catalin@gmail.com; 6 “Dr. Ion Cantacuzino” Surgery Discipline, Faculty of Medicine, “Carol Davila” University of Medicine and Pharmacy, 020021 Bucharest, Romania; gfdragos@yahoo.com; 7 Radiotherapy Department, Coltea Clinical Hospital, 030167 Bucharest, Romania; drmocanubogdan@gmail.com (B.M.);; 8 Department of Obstetrics and Gynecology, St. John Emergency Hospital, Bucur Maternity, 040292 Bucharest, Romania

**Keywords:** gynecological cancers, palliative radiotherapy, symptom relief, quality of life

## Abstract

Background: Palliative radiotherapy plays a crucial role in managing symptomatic gynecological cancers (GCs). This article aims to systematically review literature studies on palliative pelvic radiotherapy in cervical, endometrial, ovarian, vaginal, and vulvar cancers. The primary focus is centered around evaluating symptom relief, quality of life (QOL), and toxicity in order to ascertain optimal radiotherapy regimens. Methodology: For this thorough review, we mainly relied on Medline to gather papers published until November 2023. Selected studies specifically detailed symptomatology and QOL responses in palliative pelvic radiotherapy used for GCs. Results: Thirty-one studies, mostly retrospective studies and those lacking standardized outcome measures, showed varied responses. Encouraging outcomes were noted in managing hemorrhage (55%) and pain control (70%). However, comprehensively assessing overall symptom response rates and toxicity remained challenging. Investigations into 10 Gy fractionation revealed benefits in addressing tumor-related bleeding and pain in female genital tract cancers. Conclusions: Palliative pelvic radiotherapy effectively manages symptomatic GCs. Nonetheless, unresolved dosing and fractionation considerations warrant further investigation. Embracing modern therapies alongside radiotherapy offers improved symptom control, emphasizing the importance of selecting suitable patients for successful GC palliation interventions.

## 1. Introduction

Gynecological cancers are a significant global health concern, contributing to cancer-related mortality and profound suffering [1]. Late-stage presentation often results from inadequate screening and limited access to medical care [2]. Cervical cancer, the fourth most prevalent cancer in women globally, is associated with heightened pain, anxiety, and depression [3]. Similarly, vulvar cancer, although less common, also poses substantial psychological challenges to affected individuals, as highlighted by recent research. Additionally, endometrial, ovarian, and vaginal cancers contribute to the burden of gynecological malignancies, each presenting its unique set of symptoms and challenges. Patients with these cancers endure distressing symptoms, such as vaginal discharge, bleeding, sexual impairment, and financial strain, often leading to a high rate of partner abandonment [4].

Palliative treatment, including palliative radiotherapy, plays a crucial role in alleviating symptoms and improving the quality of life for patients with advanced gynecological cancers. It focuses on symptom management, pain relief, and enhancing overall comfort rather than curative intent. The decision to initiate palliative radiotherapy is influenced by various factors, including the patient’s general status and expected survival time. For patients with limited life expectancy or poor general health, palliative radiotherapy can offer significant symptom relief and improve quality of life, even in the absence of curative potential. Early qualification for palliative radiotherapy ensures timely symptom control and may positively impact patient outcomes by minimizing distress and enhancing comfort during the advanced stages of the disease [5,6,7,8].

Palliative care integrates various approaches, including radiotherapy, medical therapies, nerve blocks, surgery, and psycho-oncology, to address the multifaceted distress experienced by patients [5,6]. Neuropathic pain and hemorrhage are common due to the proximity of nerves and rich blood supply to pelvic malignancies [7]. Palliative radiotherapy effectively alleviates symptoms, with hypofractionation emerging as a cost-effective and minimally toxic option [8]. This systematic review aims to identify and analyze published studies that elucidate the impact of palliative pelvic external beam radiotherapy (EBRT) on symptomatic gynecological cancers (cervix, uteri, vagina, vulva, and ovary cancers). This review intends to offer clarity regarding its effect on pelvic symptoms, quality of life (QOL), and toxicity profiles, while also attempting to evaluate treatment schedules. The ultimate goal is to discern if an optimal dose or fractionation regimen exists and establish whether any correlation exists with its effects. The subsequent sections will expound upon the methodology, findings, and implications derived from this systematic review.

## 2. Materials and Methods

We meticulously adhered to the guidelines outlined in the Preferred Reporting Items for Systematic Reviews and Meta-Analyses (PRISMA) statement [9] while conducting this comprehensive analysis on palliative pelvic radiotherapy in the context of gynecological cancers. The PRISMA guidelines were pivotal in structuring our systematic review, ensuring methodological rigor and transparency throughout the research process. These guidelines provided a standardized framework that helped to mitigate potential biases and enhanced the reliability of our findings.

By acknowledging the intrinsic limitations inherent in the available publications concerning palliative pelvic radiotherapy used for gynecological cancers, we took considerable care in addressing these constraints within the scope of our analysis. Our stringent adherence to the PRISMA guidelines enabled a careful evaluation of existing research while acknowledging and accounting for the limitations and gaps in the current body of literature. By following these established guidelines, we aimed to present a thorough and comprehensive review that encapsulated the available evidence in this domain while being transparent about the challenges and constraints faced in interpreting and synthesizing the data.

**Figure 1:**
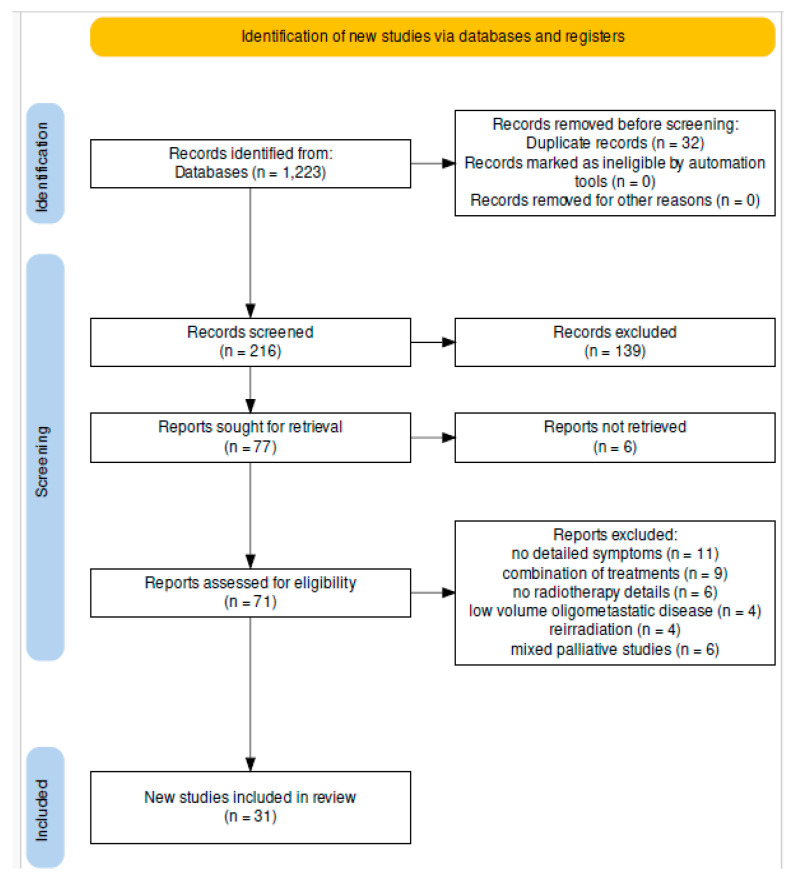
The components of the research protocol for the identification of studies via online databases and registers.

Moreover, to ensure the transparency and reproducibility of our research methodology, a detailed scientific research protocol was data presented in Figure 1. This protocol outlines the systematic approach, inclusion criteria, search strategies, and methodologies employed in our study, offering readers comprehensive insights into the framework utilized for this systematic review. It serves as a valuable resource for readers, enabling a clear understanding of the systematic process adopted to assess and synthesize the available literature on palliative pelvic radiotherapy in gynecological cancers.

### 2.1. Search Strategy

Searches in the Medline library database were performed throughout November 2023. The following MESH terms illustrate the search strategy used in Medline: (“Pelvic Neoplasms/radiotherapy” [Mesh]) OR “Uterine Cervical Neoplasms/radiotherapy” [Mesh] OR “Ovarian Neoplasms/radiotherapy” [Mesh] OR Genital Diseases, Female [MeSh] AND (palliative care [MeSH Terms]). The resultants’ titles/abstracts were screened by 3 authors (BA, SS, and BM). In addition to automated searches, a manual search was conducted to further refine the results and capture potential relevant articles missed by the initial search strategy (SS). The search strategy was refined based on insights gained from initial results, incorporating synonyms, related terms, and advanced search techniques to build a smarter query. Studies were identified by their English title (used in database indexing and reference lists) and only studies with full text available in English for review were included in our final set for analysis. Studies identified in the initial search underwent an initial screening of abstracts using a two-person review. After a two-person consensus was achieved, based on the reporting of symptoms’ outcomes and the use of radiotherapy as a treatment in the abstract, the selected titles underwent full-text review and data collection.

### 2.2. Eligibility Criteria

The analysis encompassed a comprehensive review of full-text studies focusing on pelvic external beam radiation therapy (EBRT) for gynecological cancers, specifically administered with palliative intentions. The inclusion criteria primarily emphasized studies that reported symptoms or quality of life (QOL) outcomes stemming from the palliative EBRT administered to address these gynecological malignancies. Notably, studies that combined palliative EBRT with other interventions specifically directed at targeting the tumor were excluded from consideration.

This stringent selection process sought to ensure a focused evaluation specifically centered around the effectiveness and impact of pelvic EBRT in managing symptoms and enhancing the quality of life in patients with gynecological cancers. By excluding studies involving concurrent interventions aimed directly at tumor control or management, the analysis aimed to provide a clear and distinct understanding of the role and outcomes associated specifically with palliative EBRT in alleviating symptoms and improving the quality of life for individuals afflicted by gynecological cancers.

### 2.3. Evaluation of Studies

Following a thorough qualitative evaluation aimed at assessing the internal validity of individual studies, an extensive examination was conducted to evaluate potential biases at both study and outcome levels. Notably, it is pertinent to highlight that the absence of a standardized and universally validated tool for ascertaining the “quality” of studies posed a challenge in this evaluation process. Consequently, the assessment and determination of study quality relied on a meticulous scrutiny of full-text articles, diligently performed by the authors (BA, SS, and BM). This meticulous examination encompassed a comprehensive evaluation of various parameters and factors affecting the studies’ integrity and reliability.

The evaluation process meticulously scrutinized multiple facets of the studies under consideration, emphasizing internal validity and the overall robustness of the methodologies employed. Various components, including study design, participant selection criteria, outcome measurements, and the potential impact of biases, were critically evaluated to gauge the reliability and credibility of the findings presented within the studies.

Furthermore, an essential aspect of the evaluation pertained to assessing the risk of bias inherent at both study and outcome levels. Despite the absence of a universally accepted and standardized tool for assessing study quality, the authors rigorously examined potential biases that might influence the reliability and credibility of the study outcomes. Factors such as selection bias, performance bias, detection bias, attrition bias, and reporting bias were meticulously scrutinized to ascertain the overall quality and reliability of the studies.

Ultimately, the process of final study selection was underpinned by a comprehensive and meticulous evaluation conducted by the authors, encompassing an in-depth analysis of the methodological soundness, potential biases, and overall reliability of the findings. The collaborative approach adopted by the authors, wherein consensus was achieved through collective scrutiny and discussion, ensured a thorough and robust selection of studies for inclusion in the analysis.

### 2.4. Data Extraction and Management

Data processing regarding the study features and outcomes of interest like symptom response, QOL, and toxicity were extracted from the selected studies into tables. Initially, two reviewers (SS and BM) performed the data extraction process and a third reviewer (BA) was consulted to resolve the differences. Due to the heterogeneity of studies, a meta-analysis was not feasible, but the available tables were presented with trials to identify the association between the quality of included studies and their interpretation. After intensive attempts, no consensus was made to link the quality of included studies and their interpretation; therefore, a decision was made to present the data in table form.

## 3. Results

### 3.1. Study Selection

Upon initiating the search process, an initial yield of 216 records was obtained. After careful screening (involving the elimination of duplicate entries and the exclusion of abstracts that did not meet the relevancy criteria), a refined set of 77 articles, encompassing original research and systematic reviews, was subjected to meticulous examination. A comprehensive analysis was conducted on these 77 articles through a thorough assessment of their full texts, aligning with the pre-established inclusion and exclusion parameters. Following this rigorous evaluation, 31 carefully selected studies that met the predetermined criteria were ultimately included for the final scrutiny and synthesis (as illustrated in Figure 1). These studies specifically delved into the utilization of external beam radiotherapy (EBRT) or brachytherapy (BT) as modalities for palliative intervention in gynecological cancers.

### 3.2. Study Characteristics

Table 1 furnishes a comprehensive snapshot of the 31 studies that were incorporated into this analysis. These studies exhibited a median participation count of 44 individuals (ranging from 2 to 184 participants), cumulatively enrolling a total of 1365 patients from diverse retrospective cohorts. Among these studies, three were prospective investigations that spanned treatment periods from 1983 to 2020. Despite the valuable insights gleaned from these studies, a notable observation was the lack of standardized scales or uniform methodologies used for capturing patient-reported outcomes or symptom-related data across the reviewed literature. This absence of standardized measures for assessing patient-reported outcomes and symptomatology was apparent in the range of studies scrutinized, which potentially impacts the comparability and consistency of the reported findings.

### 3.3. Patient Characteristics and Symptoms

The population represented in the included studies exhibited a diverse array of characteristics, showcasing notable heterogeneity across various parameters. Previous studies [4] accentuate the prevalence of symptoms reported, emphasizing the prominence of manifestations related to bleeding, discharge, and pain as the most frequently reported symptoms. Furthermore, indications for therapeutic intervention were not limited solely to the primary presenting symptoms but also encompassed rectal and ureteral manifestations stemming from external pressure or localized invasion based on the malignancy. Interestingly, in certain cases, the treatments administered were not solely focused on relieving immediate symptoms but also aimed at locally controlling the tumor or hindering its progression [10]. This diversity in treatment goals and the spectrum of symptoms that were addressed highlights the multifaceted nature of symptomatology in gynecological cancers and underscores the complexity in managing these conditions comprehensively.

### 3.4. Radiotherapy Dose and Fractionation

The treatment strategies employed in these studies primarily centered on fractionation schemes consisting of 2–3 Gy delivered daily, exhibiting variations in radiotherapy doses, techniques, and schedules, along with a diverse range of target definitions. The majority of authors documented the usage of both linear accelerators and cobaltotherapy units, highlighting their widespread adoption, in addition to mentioning the utilization of BT units. A subset of studies opted for hyperfractionation, employing fractionation of less than 2 Gy (delivered twice daily) in an attempt to impede the progression of the disease. The total radiation doses administered varied significantly, spanning from 8 to 76 Gy (refer to Table 1), with hypofractionation emerging as the more commonly preferred approach.

Interestingly, nearly half of the studies (14 out of 31) included a mixed population comprising various gynecological cancers, while approximately one-third of the patient cohort had ovarian cancer as their primary malignancy. However, the absence of uniformity in the prescribed doses and treatment regimens posed a challenge in establishing a biologically equivalent dose across these studies. The heterogeneous nature of the treatments employed underscores the lack of standardized approaches in palliative radiotherapy used for gynecological cancers, necessitating further exploration and consensus to enhance treatment efficacy and patient outcomes. For example, the study by Benoîte Méry et al. [10] comprises 19 patients (aged 90 to 98.6 years) treated with radical irradiation (likely employing a dose of 76 Gy in this subgroup), raising doubts about its applicability to the broader population of patients undergoing palliative radiotherapy. A careful consideration of such outliers is crucial in interpreting the findings and ensuring the generalizability of the systematic review’s conclusions.

### 3.5. Treatment Response

The definition of response criteria lacked a consensus across the diverse range of studies that were analyzed. The term “effective palliation” was commonly employed in conjunction with descriptors like “symptomatic relief” or “control”. A notable proportion (17 out of 31 studies) reported achieving bleeding control without specifying the effects of the duration or providing comprehensive treatment follow-up. Likewise, significant success in managing pain was evident in 22 out of the 31 studies. However, only a limited number of studies, specifically four, reported instances of remission in vaginal discharge.

### 3.6. Durability of Response

Among the varied symptoms documented, vaginal hemorrhage appeared as an early-responding symptom in several instances, sometimes resolving during the course of treatment. Most studies defined a positive response as the alleviation of symptoms or the achievement of effective palliation. However, certain standout studies presented unique outcomes. For instance, a study that focused on reirradiation utilizing high-dose interstitial brachytherapy for locally recurrent cervical cancer showcased a commendable 76.9% local control rate and a median post-recurrence survival period of 32 months, accompanied by a toxicity profile of 25% graded between 3 and 4 [11]. Similarly, another study illustrated remarkable long-term palliation in the oligometastatic setting of ovarian cancer, attaining high local control through metastasis-directed stereotactic body radiotherapy (SBRT) without inducing significant irradiation-related toxicities [12]. Despite the array of symptoms reported, establishing a median duration of symptomatic relief posed challenges across the spectrum of studies analyzed.

### 3.7. Dose Response

Establishing a direct relationship between the administered dose of radiotherapy and the response observed within the studies proved to be challenging. Despite this challenge, an observable trend emerged, suggesting that a higher radiotherapy dosage correlated with enhanced local control or symptom relief, as evidenced by the trends outlined in Table 1. In specific instances, hypofractionation or repeated fractionation demonstrated efficacy in addressing bleeding symptoms, showcasing potential benefits. For instance, a study conducted by Tonny Snijders-Keilholz et al. delineated suboptimal outcomes in cases treated with palliative intent. Their findings suggested that contemplating more aggressive treatment strategies might be prudent for achieving enhanced local control and potentially improved survival rates [13].

### 3.8. Toxicity

The comprehensive assessment of radiation treatment toxicity profiles across most studies was inconsistent, with 12 of them suggesting minimal or negligible levels of toxicity. There were attempts in some studies to systematically grade the toxicity related to palliative radiotherapy. Describing low toxicity when minimal clinical effects or interventions were necessary, moderate toxicity when treatments were temporarily halted due to complications, and high or severe toxicity leading to treatment cessation provided a general framework for categorizing adverse effects.

However, the detailed recording of toxicity profiles remained limited across the majority of studies. Only a minority (three studies) presented toxicity data for over two-thirds of the patients, explicitly indicating instances of “severe” toxicity or the necessity to discontinue treatment due to toxicity concerns. Notably, these cases involved the utilization of ultra-hypofractionation, administering a dosage of 10 Gy per treatment, either as a single application or in repeated treatments. In instances where grade 3 adverse events were reported, symptoms such as diarrhea, vomiting, and fatigue were frequently reported.

**Table 1:** Characteristics of studies on palliative pelvic radiotherapy of gynecological cancers.

First Author and Year of Publication	Study Design	Number of Patients	Gynecological Cancer	RT Dose	Number of Fractions	Dose Per Fraction	Outcome	Follow-Up	Toxicity
Faul et al., 2000 [14]	Observational prospective	2	Ovarian	7	1	7	100% complete response (bleeding control) at 1 month	n/a	n/a
Macchia et al., 2016 [15]	Observational prospective	9	Cervical and uterine	30	3	10	89% CR; 11% marked improvement (bleeding control)	20 months	Low
Georgina L Jones et al., 2006 [16]	Observational prospective	16	Ovarian	n/a	n/a	Single fraction of 7 Gy or 2 fractions of 3 Gy b.d.	Effective palliation, pain relief, and symptom relief	3 months	n/a
F L Ampil et al., 2006 [17]	Retrospective observational	79	Cervical	n/a	n/a	n/a	Tumor control	11 months	Moderate
Benoîte Méry et al., 2016 [10]	Retrospective observational	19	Uterine, cervical, vulvar, and vaginal	Median of 45 Gy (range: 6–76 Gy)	Median of 18 (range: 1–36 fractions)	Median of 3 Gy (range: 1.5–6 Gy)	Tumor control	4.5 months	n/a
A Tinger et al., 2001 [18]	Retrospective observational	80	Ovarian	n/a	n/a	n/a	Partial response	60 months	Moderate
Sri Harsha Kombathula et al., 2022 [19]	Retrospective observational	184	Cervical, vaginal, uterine, and ovarian	Median of 35 Gy (range: 10–50 Gy)	Median of 15 (range: 1–20)	Median of 2.33 Gy (range: 2.33–10)	Symptom relief	36 months	n/a
Boulware et al., 1979 [20]	Retrospective observational	86	Cervical, vaginal, uterine, and ovarian	10 Gy	1	10 Gy	Bleeding control and pain relief	6 months	Low
Boulware et al., 1979 [20]	Retrospective observational	55	Cervical, vaginal, uterine, and ovarian	10 Gy at 3–4-week interval	1	10 Gy	Bleeding control and pain relief	6 months	Moderate
Boulware et al., 1979 [20]	Retrospective observational	20	Cervical, vaginal, uterine, and ovarian	10 Gy at 3–4-week interval	1	10 Gy	Bleeding control and pain relief	6 months	n/a
Hodson et al., 1983 [21]	Retrospective observational	27	Cervical, vaginal, uterine, and ovarian	10 Gy at 3–4-week interval	1	10 Gy	Bleeding control, pain relief, and improved vaginal discharge	7 months	Low
Halle et al., 1986 [22]	Retrospective observational	42	Cervical and uterine	10 Gy at 3–4-week interval	1	10 Gy	Bleeding control, pain relief, and improved vaginal discharge	10 months	Low
Onsrud at al., 2001 [23]	Retrospective observational	64	Cervical and uterine	10 Gy	1	10 Gy	Bleeding control and improved vaginal discharge	12 months	low
Mishra et al., 2005 [24]	Retrospective observational	100	Cervical	10 Gy at 4 weeks; brachytherapy 30 Gy at point A	1–3	10 Gy	Bleeding control, pain relief, and improved vaginal discharge	9 months	High
Patricio et al., 1987 [25]	Retrospective observational	56	Cervical	13 Gy	2	6.5 Gy	Bleeding control and pain relief	n/a	High
Spanos et al., 1996 [26]	Subgroup analysis of a prospective trial	61	Cervical	14.8 Gy	4	3.7 Gy b.d.	Bleeding control and pain relief	12 months	Low
Grigsby et al., 2002 [27]	Retrospective observational	15	Cervical	10 Gy	2	5 Gy	Bleeding control	n/a	No toxicity
Choan E. et al., 2006 [28]	Retrospective observational	53	Ovarian	Median of 30 Gy (range: 5–52.5 Gy)	Median of 10 Gy (range: 1–20)	Median of 3 Gy (range: 2.62–5 Gy)	Bleeding control and pain relief	n/a	Low
M D Adelson et al., 1987 [29]	Retrospective observational	42	Ovarian	10–30 Gy	1 to 3	10 Gy	Bleeding control and pain relief	n/a	High
Corn et al., 2001 [30]	Retrospective observational	33	Ovarian	35 Gy (range: 7.5–45 Gy)	n/a	Median of 2.5 Gy (range: 1–5 Gy)	Symptom relief, bleeding control, pain relief	n/a	Low

## 4. Discussion

### 4.1. Symptom Control in Gynecological Cancers

Palliative radiotherapy exhibits substantial promise in controlling symptoms associated with gynecological cancers. As summarized in Table 2, studies demonstrate varying success rates in alleviating symptoms like bleeding, pain, and vaginal discharge across different treatment regimens [14,15,18,20,22,23,24,25,27,30,31,32,33,34,35,36].

The evolution of radiation therapy techniques has ushered in new avenues for managing gynecological malignancies, particularly in cases of recurrent disease where conventional treatments may have been exhausted. Studies have explored the feasibility of reirradiation using high-dose-rate interstitial brachytherapy for locally recurrent cervical cancer, shedding light on its efficacy within a single institutional context [11]. Additionally, investigations have delved into salvage radiotherapy strategies following postsurgical relapses of endometrial cancer, offering valuable insights into treatment modalities for recurrent disease scenarios [37]. In the pursuit of effective therapeutic options for recurrent pelvic and primary gynecologic malignancies, isolated studies examined the utilization of 241 Am, presenting potential avenues for therapeutic intervention [38]. Other research [39] contributed to this discourse by assessing the palliative benefits of external-beam radiation in managing platinum-refractory epithelial ovarian carcinoma, underscoring its role in symptom alleviation. Moreover, other investigations [40] provided valuable evidence regarding the efficacy of single-fraction palliative pelvic radiation therapy in gynecologic oncology, highlighting its potential in symptom management.

**Table 2:** Palliative pelvic radiotherapy for symptomatic advanced gynecologic cancers.

Symptom	Overall Response Rate
Bleeding	45–100% [20,21,22,23,24,30,31,41,42,43,44]
Pain	0–83% [20,21,22,23,24,45]
Discharge	39–49% [20,23,24]
Obstruction	19–100% [18,20,41,45]

For instance, studies adopting normo-fractionation or hyperfractionation approaches have reported high control rates for bleeding, albeit with smaller sample sizes [18]. However, it is important to note that specific details regarding dose fractionation were not available in Table 1 for Reference [18]. Furthermore, conventional fractionation was employed in studies like [32,43] which also reported favorable outcomes in bleeding control. Conversely, hypofractionation EBRT techniques have shown controlled bleeding in approximately 93% of cases with varying median total doses [28,30,33]. However, weekly treatments using hypofractionation have been found to exhibit lower rates of bleeding relief [20,22,23,25,31]. High-dose-rate brachytherapy (HDR-BT) has emerged as a valuable option, reporting partial bleeding control rates exceeding 85% [24,27,34,35,36]. A study focusing solely on BT revealed that 93% of subjects experienced controlled bleeding without further interventions [27].

The diversity in fractionation schemes also impacts symptom reduction. Some studies showcased the efficacy of repeated doses within weeks to control bleeding, while others adopted a single 10 Gy fraction repeated based on patient responses [20,21,22,25,26,27]. This varied approach warrants further exploration in order to ascertain the most effective regimens for different symptom presentations.

### 4.2. Toxicity Profiles and Technological Advancements

Assessing the toxicity profile associated with palliative radiotherapy presents considerable challenges owing to variations in recording methodologies and reporting practices among the studies that were reviewed. While the general trend leans towards the observation of low or minimal toxicity in the majority of cases, some notable instances stand out, particularly in connection with ultra-hypofractionation, where severe adverse events were reported [46,47,48,49,50].

The evolution of technology in radiation planning and delivery has introduced promising avenues aimed at mitigating radiation-induced toxicities [51,52]. For instance, the implementation of high-energy photons coupled with CT-based techniques represents a significant advancement. These technological improvements play a pivotal role in refining dose precision while simultaneously reducing exposure to adjacent healthy tissues [53,54]. Such refinements are critical in alleviating the adverse effects typically associated with radiotherapy, thereby enhancing patient outcomes and comfort during treatment.

Another potential avenue for improving therapeutic outcomes involves the integration of radiotherapy with radiosensitizing agents. While this approach holds promise in enhancing the effectiveness of radiotherapy, its full potential remains underexplored and demands further comprehensive exploration in prospective studies. Understanding the synergistic effects and potential interactions between these agents and radiation therapy is vital in order to ascertain their true impact on therapeutic efficacy and toxicity levels.

The prospect of combining radiotherapy with radiosensitizing agents introduces a potential paradigm shift in treatment approaches. However, this direction necessitates robust prospective investigations that are carried out to elucidate the safety, efficacy, and long-term implications of such combinations in clinical practice. These studies should encompass a meticulous evaluation of both therapeutic benefits and associated toxicities in order to establish a comprehensive understanding of the potential advantages and limitations of this treatment modality in the context of gynecological cancers.

### 4.3. Clinical Applications and Challenges

Radiotherapy assumes a pivotal role in managing symptoms associated with gynecological cancers, offering an essential avenue for symptomatic relief in affected individuals. The effective application of palliative radiotherapy hinges upon a thorough evaluation encompassing several crucial factors, including patient prognosis, the intensity of symptom burden, performance status, and adherence to treatment protocols [55]. These factors collectively shape the treatment landscape and guide the formulation of a tailored therapeutic strategy personalized to each patient’s unique circumstances.

However, the translation of optimal dose fractionation schedules or treatment approaches from theoretical frameworks to real-world clinical settings presents multifaceted challenges. These challenges are multifaceted and require comprehensive consideration. They span various domains, including clinical, logistical, and patient-centered aspects. Overcoming these challenges necessitates a nuanced patient-centric approach that accounts for the intricate interplay between different factors influencing treatment efficacy, toxicity profiles, and patient adherence.

One primary challenge involves aligning the prescribed treatment regimen with individual patient characteristics and disease presentations. Tailoring treatment approaches demands a comprehensive understanding of patient-specific variables, such as disease stage, tumor characteristics, and the overall health status of the individual. This tailored approach enables healthcare providers to optimize treatment outcomes while minimizing the risk of undue toxicity.

Additionally, navigating the complexities associated with treatment compliance and patient tolerance poses substantial hurdles in real-world clinical scenarios. While a theoretical treatment plan might appear optimal on paper, the actual administration and adherence to the prescribed regimen in real-world settings could encounter various obstacles. Factors such as patient accessibility to healthcare facilities, socioeconomic considerations, and potential treatment-related adverse effects can significantly impact treatment adherence and outcomes.

To address these challenges effectively, a holistic and patient-centered approach is imperative. This approach should encompass multidisciplinary collaboration among healthcare providers, including oncologists, radiation therapists, nurses, and allied healthcare professionals, in order to develop personalized treatment strategies. Moreover, patient education and support programs play a pivotal role in ensuring treatment comprehension, enhancing adherence, and mitigating potential treatment-related anxieties or misconceptions.

Ultimately, the effective implementation of optimal dose fractionation schedules and treatment approaches in the clinical setting necessitates a multifaceted approach that acknowledges the intricate interplay between clinical considerations, logistical challenges, and patient-centered care. A tailored approach that accounts for individual patient needs and characteristics remains fundamental in optimizing treatment efficacy while ensuring patient comfort and adherence to therapy.

### 4.4. Limitations

Despite the comprehensive analysis presented in this systematic review, several limitations must be acknowledged. Firstly, the inclusion of specific studies available in English and the exclusion of non-English publications may introduce language bias, with relevant research conducted in other languages being potentially overlooked. Additionally, while efforts were made to ensure a thorough search strategy, including manual searches and screening carried out by multiple reviewers, it is possible that some relevant studies may have been missed. Furthermore, the heterogeneity in the included studies, particularly in terms of patient populations, treatment regimens, and outcome measures, limits the ability to draw definitive conclusions. The variability in reporting standards and the lack of standardized assessment tools for symptom relief and toxicity further complicate the synthesis of results. Finally, the retrospective nature of many studies included in this review may introduce inherent biases and limitations inherent to retrospective data analysis, including selection bias and incomplete data capture. Despite these limitations, this systematic review provides valuable insights into the current state of knowledge regarding the use of palliative radiotherapy in gynecological cancers and highlights areas for future research and improvement.

### 4.5. Future Research and Implications

The results of this systematic review hold significant implications for future research directions, treatment strategies, and healthcare policies in the management of gynecological cancers.

Firstly, the findings underscore the need for further research to validate and refine optimal fractionation schedules and treatment approaches. Future studies should focus on conducting prospective investigations in order to establish the most effective radiation dose and fractionation regimens for different gynecological cancer types and symptom presentations. Additionally, exploring the synergistic effects of systemic therapies in conjunction with radiotherapy presents a promising avenue for enhancing treatment responses and improving outcomes for patients.

In terms of treatment strategies, the diverse approaches to palliative radiotherapy highlighted in this review suggest the importance of personalized treatment plans tailored to individual patient characteristics and disease presentations. Clinicians should consider factors such as disease stage, tumor characteristics, and patient performance status when formulating treatment strategies that optimize outcomes while minimizing toxicity.

From a healthcare policy perspective, these findings emphasize the need for standardized reporting standards and the development of validated tools used for assessing symptom relief in gynecological cancer patients undergoing palliative radiotherapy. Establishing uniform guidelines for treatment planning and delivery could help standardize care practices and improve consistency in patient outcomes across different healthcare settings.

Overall, the results of this systematic review provide valuable insights that can inform future research endeavors, guide the development of personalized treatment strategies, and influence healthcare policies aimed at improving the quality of care for patients with gynecological cancers.

## 5. Conclusions

In conclusion, this systematic review underscores the pivotal role of palliative radiotherapy in managing symptoms associated with gynecological cancers. Our analysis revealed the effectiveness of various treatment regimens, including normo-fractionation, hyperfractionation, hypofractionation, and HDR-BT, in mitigating symptoms such as bleeding, pain, and vaginal discharge. However, the lack of a consensus in treatment approaches, dose fractionation schedules, and reporting standards presents challenges in standardizing optimal strategies used for symptom control.

Moving forward, it is imperative to address these challenges and capitalize on the opportunities identified in this review. By emphasizing the significance of the findings, particularly the efficacy of different fractionation schemes and the potential of HDR-BT, we can predict future research directions and guide clinical practice. Prospective studies are warranted in order to validate the most effective fractionation schedules and explore the synergistic effects of systemic therapies in conjunction with radiotherapy. Additionally, investigating the role of advanced radiation techniques and incorporating radiotherapy with radiosensitizing agents hold promise in improving therapeutic outcomes.

Furthermore, enhancing the standardization of reporting standards and developing validated tools for assessing symptom relief are essential steps in advancing this field. By bridging existing gaps and refining treatment approaches, we can optimize patient outcomes and establish standardized protocols. Ultimately, this comprehensive review contributes to our understanding of palliative radiotherapy in gynecological cancers and lays the groundwork for future research endeavors aimed at improving the quality of care for affected individuals.

## Data Availability

All data are available upon request from the corresponding author.
